# Spinal Cord Transection in a Type II Odontoid Fracture From a Ground-Level Fall

**DOI:** 10.7759/cureus.12342

**Published:** 2020-12-28

**Authors:** Samiat Agunbiade, Patrick J Belton, Fassil B Mesfin

**Affiliations:** 1 Neurosurgery, University of Missouri School of Medicine, Columbia, USA

**Keywords:** odontoid fracture, spinal cord transection, ground level fall

## Abstract

Odontoid fractures typically occur as a result of trauma: high-velocity injuries like motor vehicle accidents in young people and falls for the elderly. Odontoid fractures are the most common cervical spine fractures in patients over 65, with type II being the most common. However, spinal cord transections are rare with these types of injuries, especially without significant fracture displacement, translation or evidence of ligamentous disruption on post-injury imaging. We report a case of a patient who sustained a spinal cord transection secondary to an acute type II odontoid fracture from a ground-level fall, without computed tomography radiographic evidence of cord disruption or impingement.

## Introduction

Odontoid fractures account for 10-15% of cervical spine fractures, with type II (fracture through the base of the dens) being the most common, accounting for over 50%, followed by type III (fracture through the body of the dens), and type I (fracture of the tip of the dens) being exceedingly rare [1,2]. Odontoid fractures typically result from blunt trauma to the cervical spine, most commonly high-velocity injuries in younger patients, such as motor vehicle accidents, and falls in elderly patients [2,3]. Odontoid fractures are the most common cervical spine fractures in patients over 65 with type II the being most common [4,5]. Although odontoid fractures have a neurological injury rate ranging from 7.5% to 33% [5-7], cervical spinal cord injuries following odontoid fractures are rare [6], especially without significant translation, displacement or ligamentous disruption. This is likely to the relatively large cross-sectional diameter of the spinal canal at that odontoid level, compared to the diameter of the spinal cord [1]. We report a case of a patient who sustained a spinal cord transection following a type II odontoid fracture from a ground-level fall.

## Case presentation

The patient was a 78-year-old female with a past medical history of type II diabetes mellitus who was found unresponsive by her family following a ground-level fall. The patient was carrying groceries and collapsed or tripped, striking her face. The event was witnessed by one of her daughters. Her daughter reported that on rushing to her assistance she had no pulse and had turned blue. Bystanders performed cardiopulmonary resuscitation (CPR) while waiting for medical assistance. When emergency medical personnel arrived, the patient was opening her eyes but did not respond to any external stimuli and was subsequently was intubated. When the patient arrived at the outside hospital, she was hypotensive with a blood pressures of 60/40 and Philadelphia type cervical collar was placed. The patient was transferred to a tertiary care center for a higher level of care.

**Figure 1:**
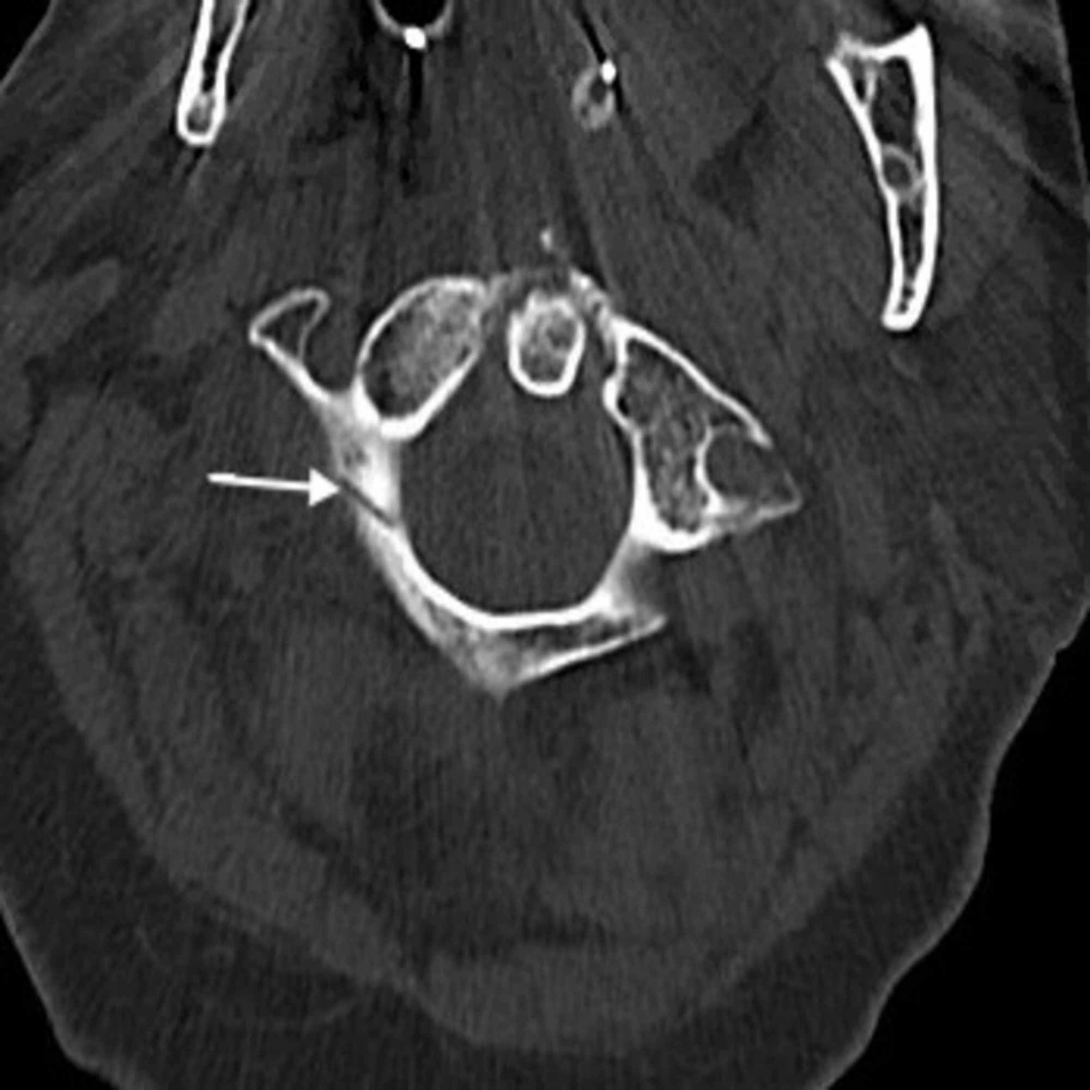
Axial CT of the cervical spine showing C1 posterior arch fracture. There is an acute non-displaced C1 arch fracture with bilateral transverse foramen involvement without displacement.

**Figure 2:**
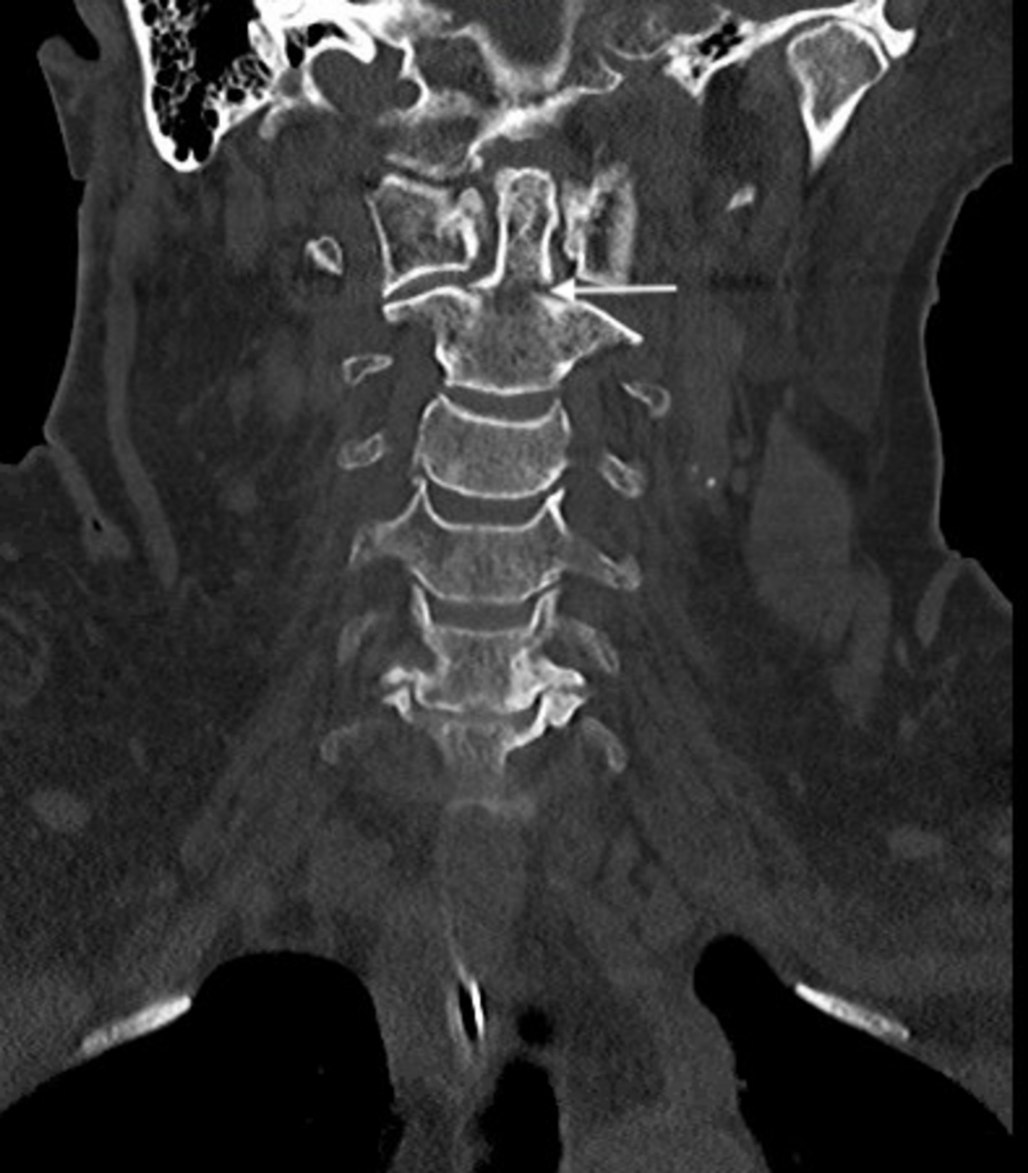
Coronal CT of the cervical spine with type II odontoid fracture. Coronal non-contrast CT bone window of type II odontoid fracture.

**Figure 3:**
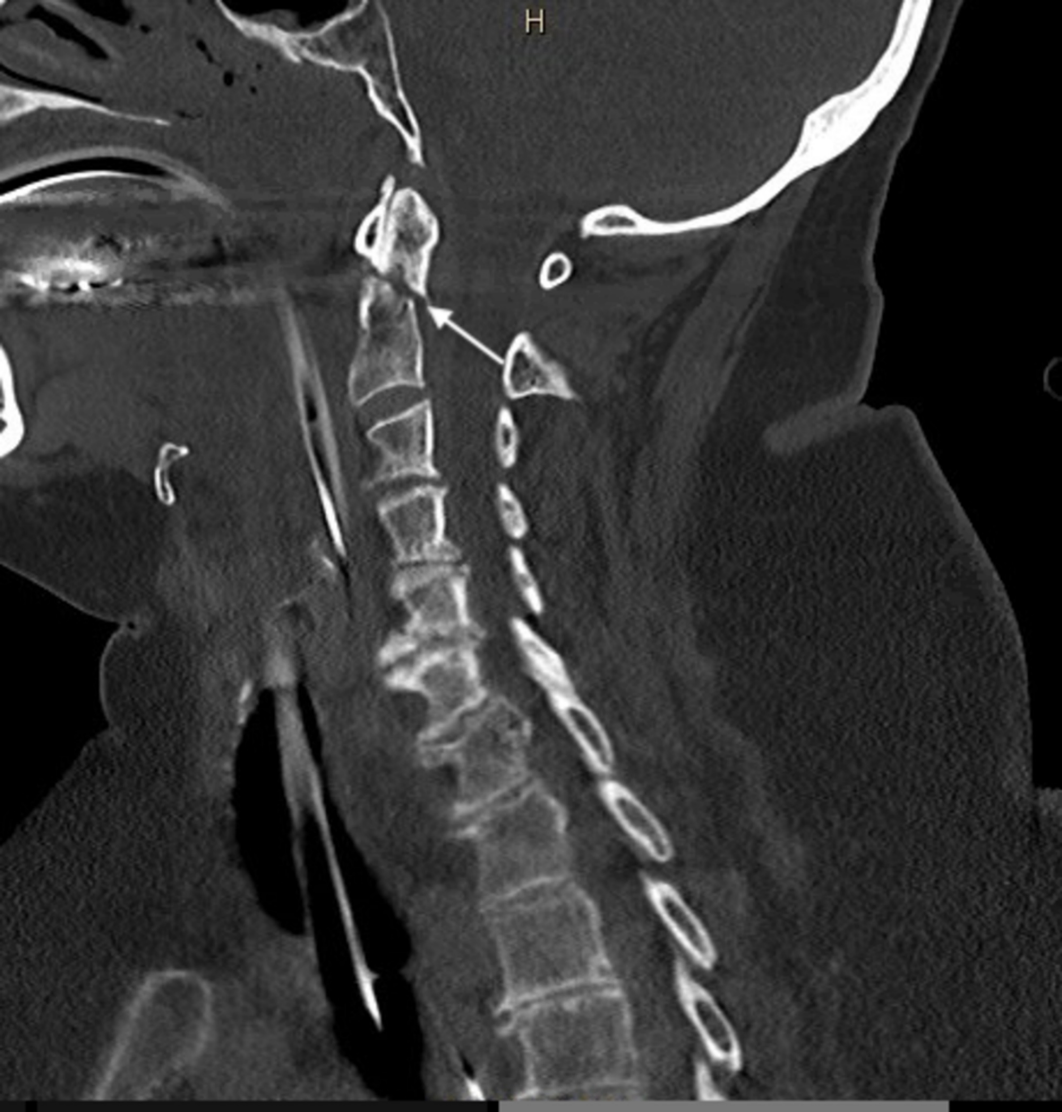
Parasagittal section of type II odontoid fracture. The parasagittal view of the odontoid fracture shows 2-3 mm posterior displacement and subluxation of C1 on C2.

On arrival at the tertiary care center, Glasgow Coma Score was 3(t). Pupils were equal and reactive to light, but sluggish, with random eye movements. She had no corneal reflex or cough reflexes, but a mild gag reflex was appreciated. There were periodic facial movements that resembled myoclonic jerks. No rectal tone was noted, but diffuse hyperreflexia was noted. Computed tomography (CT) of the brain was negative for intracranial hemorrhages, but CT of the cervical spine demonstrated a C1 posterior arch fracture (Figure 1), and acute mildly displaced type II odontoid fracture with 2-3 mm posterior displacement and mild posterior subluxation of C1 on C2 (Figures 2 and 3). Whole-body survey imaging was significant for multiple rib fractures, likely from CPR in the field.

The patient was placed in a Miami J collar and admitted to the intensive care unit for suspected anoxic brain injury. There was concern that the patient had experienced a cardiac event that caused a sudden loss of pulse. However, multiple troponins were negative and the admission electrocardiogram did not show ST elevations consistent with a cardiac event. Doppler studies showed an occlusive deep vein thrombosis in the right peroneal vein. A saddle pulmonary embolism was suspected to have caused a syncopal episode and cardiac arrest; the patient was started on heparin. Echocardiogram showed normal left and right ventricular size, with an ejection fraction of 55% and normal wall motion throughout. CT of the chest with pulmonary embolism protocol did not demonstrate a pulmonary embolism. An electroencephalogram was performed, and the results were consistent with severe diffuse encephalopathy, with no signs of seizure activity.

**Figure 4:**
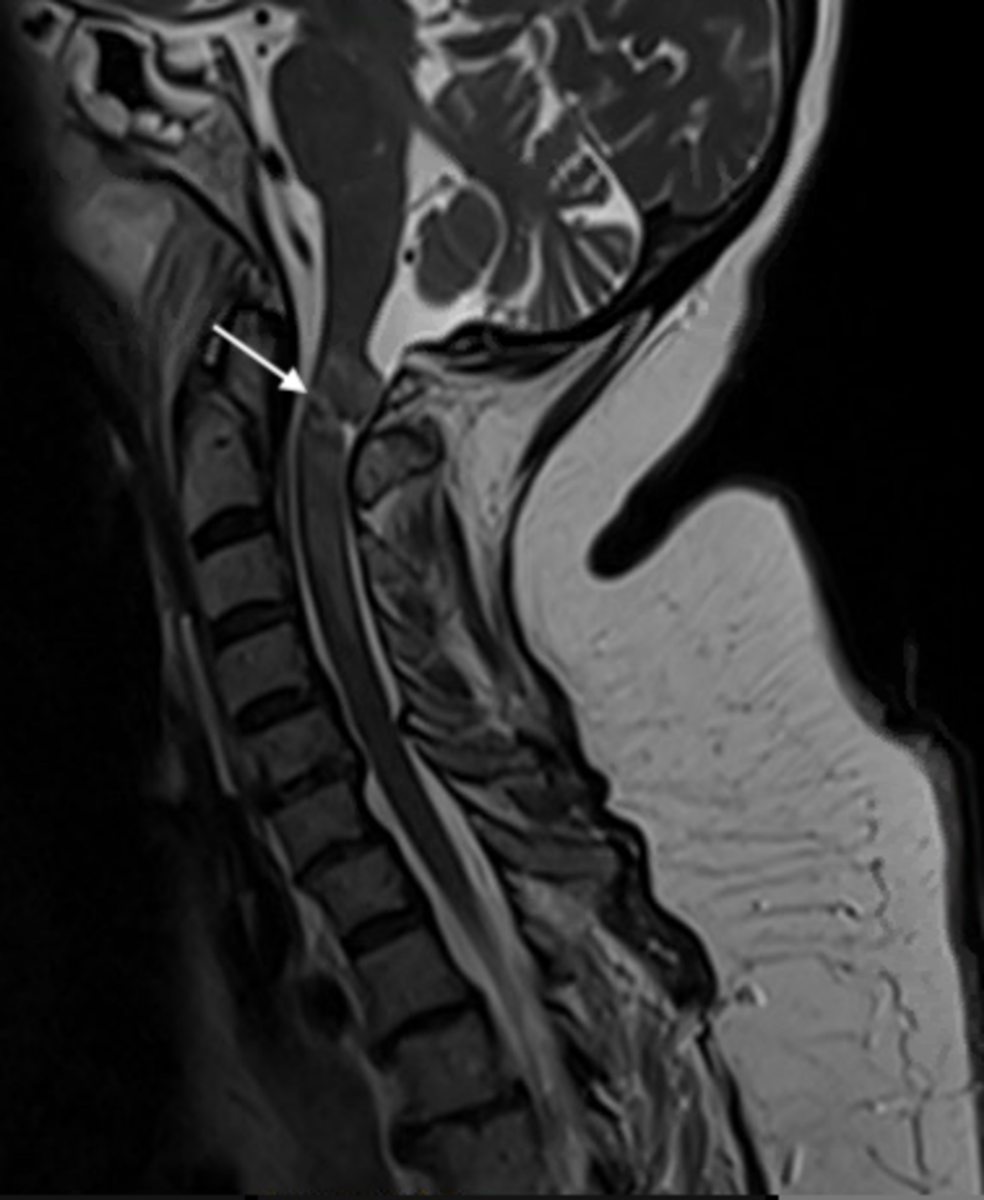
T2-weighted MRI of the cervical spine with a parasagittal view. The parasagittal view demonstrates high-grade partial spinal cord transection at the level of C1-C2. There is also an associated spinal cord contusion and a small amount of intrathecal blood.

Magnetic resonance imaging (MRI) of the brain and cervical spine were performed. On the MRI of the cervical spine, the patient was found to have a transection of the spinal cord at C1-C2 (Figure 4). Short tau inversion recovery (STIR) sequence redemonstrated the acute spinal injury without ligamentous disruption.

The patient exhibited signs of neurogenic shock, with bradycardia, hypotension, and warm extremities. The MRI results, prognosis, and the likely possibility of locked in phenomenon were discussed with the family. Ultimately the family rescinded a do not resuscitate order and elected a palliative route. The patient was extubated and passed away soon after.

## Discussion

Odontoid fractures are more commonly seen in higher velocity impacts, and although odontoid fractures in the elderly are not rare, an injury this severe, given the mechanism of a ground-level fall, is extremely unusual. For the elderly population, the odontoid fractures can occur from low energy impacts such as falls from standing positions, likely due to decreased bone density; with hyperextension of the C-spine, causing the head of C1 to be pushed backwards being the most common mechanism of injury [6-8]. Hyperflexion can also lead to odontoid fractures due to the transmission of excess forces via the transverse ligament [2]. Given the facial injury, hyperextension may have been the patient’s mechanism of injury. There may also be transverse ligament tears associated with type II fractures. Many type II fractures can mimic transverse ligament injuries with the loss of translational restriction of C1 on C2, increasing the risk of spinal cord injury and cranio-cervical deformities with poor healing [8]. However, in our patient, on MRI, there was no indication of significant ligamentous damage and displacement in her MRI. There was no indication of spinal cord compression, distraction injury, or significantly large hematoma that may have contributed to the spinal cord transection in this patient.

Traumatic cervical fractures with translation and displacement are unstable and are at increased risk of spinal cord injury [9]. However, in this patient, there was no significant displacement of the C2 fracture on survey imaging in the rigid collar, which would make the spinal cord transection that occurred in this patient even more extraordinary. The translation is defined as 3.5 mm or more of sagittal or coronal malalignment of one or more cervical vertebrae [10,11]. There have been no previously reported cases of spinal cord injuries in non-displaced fractures. The C1 fracture was nondisplaced and the odontoid fracture sustained by our patient had only a mild posterior displacement of 2-3 mm, and mild posterior subluxation of C1 on C2. We suspect the patient has had a transient displacement of the fracture, causing the spinal cord transection. Additionally, we hypothesize that the patient may have also suffered an anoxic brain as the result of the high spinal cord transection, given the myoclonic facial jerks. The C2 spinal cord transection likely led to phrenic nerve paralysis since phrenic nerve innervation occurs via C3-5 causing diaphragm paralysis, rendering the patient unable to breathe on her own and contributing to an anoxic brain injury. The patient’s hypotension was likely due to neurogenic shock from the high spinal cord injury further exacerbating her anoxic brain injury. Neurogenic shock can be seen in spinal cord injuries above T6 and is characterized by hypotension, bradycardia and autonomic dysfunction [12]. Hypotension occurs as the result of the loss of systemic vascular resistance from the loss of sympathetic tone, leading to vasodilation from unopposed parasympathetic responses [12]. Flexion force may result in airway edema, resulting in airway obstruction [13]. The patient had a normal echocardiogram and imaging did not demonstrate any signs of a pulmonary embolism, ruling out a cardiac source of hypotension. The patient was likely exhibiting signs of a locked-in syndrome as the result of her injuries.

Although spinal cord injuries following odontoid fractures are rare, there are certain factors that increase the risk of sustaining spinal cord injuries. Harrop et al. found that spinal cord injuries from odontoid fractures are more typically seen in males, with smaller spinal canals that suffer from high-velocity injuries [6]. Degenerative changes of the spinal cord such as spondylosis and ossification of the posterior longitudinal ligament and more narrow spinal canals are important preexisting factors to the risk of sustaining spinal cord injuries [14].

Many in the elderly population are at higher risk of surgical complications due to co-morbidities or poor bone quality but type II odontoid fractures are considered inherently unstable and are at increased risk of nonunion due to poor vascularity of the odontoid process [8]. There are high rates of nonunion with conservative management in elderly patients [1,5,8]; patients over 50 have a 21-fold greater incidence of nonunion with surgical management [15]. Chapman et al. in their study of 322 patients over 65 with type II odontoid fractures found that surgical treatment did not negatively impact survival; in fact, their study showed an improvement in 30-day survival and improved long-term survival with the surgical intervention [16]. Typical surgical strategies for type II odontoid fractures include direct anterior odontoid screw fixation, and posterior cervical instrumental fusion [8,17]. Nonsurgical management consists of rigid and non-rigid immobilizations with a halo or cervical collar [5,15]. The integrity of the transverse and alar ligaments also plays a significant role in determining surgical versus nonsurgical treatment, as well as the degree of anterior-posterior displacement (>5 mm) and angulation (>11°) [7,9,15].

## Conclusions

Odontoid fractures are the most common cervical spine fractures in the elderly. Severe spinal cord injuries from odontoid fractures are rare but can occur, even without significant disruption of the supporting ligaments. Spinal cord injuries should always be considered in patients with odontoid fractures. MRI imaging for spinal cord injury and ligamentous disruption should be considered in all patients with traumatic odontoid fractures and significant neurologic findings.
